# The association between extreme temperature and pulmonary tuberculosis in Shandong Province, China, 2005–2016: a mixed method evaluation

**DOI:** 10.1186/s12879-021-06116-5

**Published:** 2021-05-01

**Authors:** Dongzhen Chen, Hua Lu, Shengyang Zhang, Jia Yin, Xuena Liu, Yixin Zhang, Bingqin Dai, Xiaomei Li, Guoyong Ding

**Affiliations:** 1 Department of Epidemiology, School of Public Health, Shandong First Medical University & Shandong Academy of Medical Sciences, No.619 Changcheng Road, Taian, 271016 Shandong Province China; 2 Taian Centers for Diseases Prevention Control, Taian, 271000 Shandong Province China; 3 Shandong Center for Disease Control and Prevention, Jinan, 250014 Shandong Province China

**Keywords:** Pulmonary tuberculosis, Extreme temperature, Generalized additive model, Meta-analysis

## Abstract

**Background:**

The effects of extreme temperature on infectious diseases are complex and far-reaching. There are few studies to access the relationship of pulmonary tuberculosis (PTB) with extreme temperature. The study aimed to identify whether there was association between extreme temperature and the reported morbidity of PTB in Shandong Province, China, from 2005 to 2016.

**Methods:**

A generalized additive model (GAM) was firstly conducted to evaluate the relationship between daily reported incidence rate of PTB and extreme temperature events in the prefecture-level cities. Then, the effect estimates were pooled using meta-analysis at the provincial level. The fixed-effect model or random-effect model was selected based on the result of heterogeneity test.

**Results:**

Among the 446,016 PTB reported cases, the majority of reported cases occurred in spring. The higher reported incidence rate areas were located in Liaocheng, Taian, Linyi and Heze. Extreme low temperature had an impact on the reported incidence of PTB in only one prefecture-level city, i.e., Binzhou (RR = 0.903, 95% CI: 0.817–0.999). While, extreme high temperature was found to have a positive effect on reported morbidity of PTB in Binzhou (RR = 0.924, 95% CI: 0.856–0.997) and Weihai (RR = 0.910, 95% CI: 0.843–0.982). Meta-analysis showed that extreme high temperature was associated with a decreased risk of PTB (RR = 0.982, 95% CI: 0.966–0.998). However, extreme low temperature was no relationship with the reported incidence of PTB.

**Conclusion:**

Our findings are suggested that extreme high temperature has significantly decreased the risk of PTB at the provincial levels. The findings have implications for developing strategies to response to climate change.

**Supplementary Information:**

The online version contains supplementary material available at 10.1186/s12879-021-06116-5.

## Background

Global climate change is one of the biggest challenges which the world is facing, and the problem is the focus of the global community [1]. It is well-known that climate change threatens natural and human systems [2]. The 2020 report of the Lancet Countdown reported that climate suitability for the transmission of infectious diseases increased for dengue, malaria, and pathogenic *Vibrio* bacteria [3]. According to the assessment reports of the Intergovernmental Panel on Climate Change (IPCC), global climate change, such as climate warming and the increasing frequency of extreme weather events, has become an indisputable fact. The health effects of extreme temperature including extreme high temperature and extreme low temperature, as an extreme weather event, are complex and far-reaching. The potential public health impacts of extreme temperature can be significant and multifaceted, such as mortality [4, 5], disease of cardiovascular system [6, 7] and respiratory diseases [8, 9]. And beyond that, extreme temperature has an impact on infectious diseases, such as mumps [10], hand-foot-mouth disease [11], etc.

Pulmonary tuberculosis (PTB), as an infectious disease, threatens human health. Tuberculosis is the leading cause of death due to a single source of infection and one of the world’s top 10 causes of death [12]. Globally, an estimated 10.0 million people was attacked by tuberculosis and the incidence rate was 130 per 100,000 population in 2019. PTB remains a major health problem in China, and the incidence rate of PTB was estimated 58 per 100,000 population in 2019 [12]. As a chronic respiratory infectious disease, PTB is mainly transmitted through the respiratory tract, and epidemiological characteristics of PTB incorporate seasonal fluctuations [13]. This consequence implied that the morbidity of PTB may be influenced by meteorological factors. Some studies have shown that there is an association between PTB and meteorological variables, such as relative humidity, sunshine duration, average temperature, precipitation and average wind velocity [14, 15]. However, the association between extreme temperature and PTB is far from clear. With little research has been conducted, the effects of extreme temperature on PTB remain unknown.

This study aimed to quantify the impact of extreme temperature on PTB in Shandong Province of China, from 2005 to 2016. The results may assist with improving awareness of the impact of extreme temperatures on PTB and could help to developing strategies to fight extreme temperature and climatic change.

## Materials and methods

### Study area

Shandong Province (longitude 114°47.5′-122°42.3′E and latitude 34°22.9′-38°24.01′N), which is located in the lower reaches of Yellow River with Bohai Sea and Yellow Sea in the East with an area of approximate 155,800 km^2^ and a permanent resident population of 100.7 million, was selected as the study area. As of 2020, a total of 16 prefecture-level cities (i.e., Jinan, Qingdao, Zibo, Dezhou, Zaozhuang, Dongying, Yantai, Weifang, Jining, Taian, Weihai, Rizhao, Binzhou, Liaocheng, Linyi, and Heze), 57 municipal districts, 27 county-level cities, 53 counties and 137 county-level administrative regions in Shandong Province. In 2018, the prefecture-level city of Laiwu was abolished and incorporated into Jinan. Therefore, Laiwu was still studied as a prefecture-level city because the study period was from 2005 to 2016. In Shandong Province, there are differences quality of basic public services in different prefecture-level cities. The three cities with high quality of basic public service are Qingdao, Dongying and Jinan, while the three cities with poor quality of basic public service are Linyi, Liaocheng and Heze [16].

### Data collection and management

Data of PTB during 2005–2016 in Shandong Province were derived from the China Information System for Disease Control and Prevention. All reported PTB cases were defined based on the diagnostic criteria and principles of management for PTB (WS 288–2008) issued by Ministry of Health of the People’s Republic of China. In China, PTB is a statutory notifiable category B infectious disease. Therefore, physicians in hospitals must report every case of PTB on line within 24 h after the diagnosis. Therefore, we believe that the degree of compliance in disease notification was consistent during the study period.

Meteorological data from 2004 to 2016 were obtained from the China Meteorological Data Service Center (http://data.cma.cn). The variables for our analysis included daily average surface temperature (AST), daily average temperature (AT), daily minimum temperature (DMIT), daily maximum temperature (DMAT), daily average air pressure (AAP), daily minimum air pressure (DMIP), daily maximum air pressure (DMAP), daily average relative humidity (ARH), daily minimum relative humidity (MRH), sunshine duration (SD), daily average wind speed (AWS), daily maximum wind speed (MWS) and daily precipitation (PRE). Demographic data of Shandong Province were downloaded from the Center for Public Health Science Data in China (http://www.phsciencedata.cn/). Economic data and medical expenditure data were collected from Shandong Statistical Yearbooks 2006–2017.

According to definition of extreme temperature events by IPCC, extreme high temperature is defined as days in which the maximum temperature exceeds the 90th percentile of that day of the year, using 1961–1990 as the reference period. And extreme low temperature is defined as days at which the minimum temperature is lower than the 10th percentile of that day of the year, using 1961–1990 as the reference period. In our study, from 1981 to 2010 as the reference years, the 90th percentile of the DMAT on the same day of the same month was used as the threshold for extreme high temperature. If the DMAT from 2005 to 2016 was higher than this threshold, this day was defined as extreme high temperature. Similar to the definition of extreme low temperature, the 10th percentile of the DMIT on the same day of the same month was used as the threshold for extreme low temperature. If the DMIT from 2005 to 2016 was below this threshold, this day was defined as extreme low temperature.

### Study design and statistical analysis

A method combing a generalized additive model (GAM) and meta-analysis was adopted to carry out the risk assessment on PTB caused by extreme temperature events.

Firstly, temporal and spatial distributions, which reported morbidity of PTB of 17 prefecture-level cities in Shandong Province from 2005 to 2016, were described. The thematic map of the average annual reported incidence rate of PTB was produced by the ArcGIS 10.4 software. The average annual reported incidence rate was graded by Natural Break (Jenks), which was divided into 4 levels. The Natural Break (Jenks) can identify break points by picking the class breaks that best group similar values and maximize the differences between classes [17]. Due to the lack of meteorological data in Taian, Zaozhuang and Laiwu, the latter analyses were aimed at the remaining 14 prefecture-level cities.

Secondly, multi-collinearity might lead to multi-collinearity bias in the results, therefore, Spearman correlation coefficients were calculated between the meteorological variables to avoid the effects of multi-collinearity in meteorological variables. If the absolute value of *r* > 0.7, one of the two variables was retained by consulting the literature and considered in the GAMs [18]. Subsequently, we conducted cross-correlation analysis to explore the lag time between the daily reported incidence rate of PTB and various meteorological factors in each prefecture-level city. One study showed that the correlation between tuberculosis incidence and meteorological factors with a 3-months lag had the best goodness of fit [19], so the maximum lag time for cross-correlation analysis was set to 90 days. The lag days when the cross-correlation coefficient (CCF) was the largest and the CCF was more than double standard error (SE) were used as the optimal lag time for PTB [20], which was then included in further regression analysis.

The GAM is an extension of generalized linear model, which has a wider scope of application, and it handles the complex nonlinear relationship between dependent and independent variables through the form of functions, so that it has great flexibility [21]. GAMs with quasi-Poisson distribution family were used to explore the association between extreme temperature and PTB in our study. A penalized smoothing spline was used to adjust long-term, gross domestic product (GDP) per capita, annual medical expenses per capita and meteorological factors which were selected by Spearman analysis. “Season” was used as categorical variable to control for seasonal trends. Degrees of freedom were determined by generalized cross-validation (GCV). The model was defined as follows:

$$ {\displaystyle \begin{array}{l}\mathrm{Log}\left({Y}_t\right)=\mathrm{Log}\left(\mathrm{population}\right)+\mathrm{season}+\mathrm{extreme}\ \mathrm{temperature}+s\left(\mathrm{time}\right)+\\ {}s\left(\mathrm{meteorological}\ \mathrm{variables}\right)+s\left(\mathrm{medical}\right)+s\left(\mathrm{GDP}\right)\end{array}} $$

where *Y*_*t*_ was the daily number of reported PTB cases in time *t*. The Log (population) was used in order for the model suitable for reported incidence rate data. Extreme temperature was defined as a categorical variable, with 0, 1 and 2 representing normal temperature, extreme low temperature and extreme high temperature, respectively. Season included spring, summer, autumn and winter, with summer as the reference season. *s* (meteorological variables) was applied for adjust the impact of potential meteorological variables confounding. *s* (time), *s* (medical) and *s* (GDP) were used to control long-term effect, annual medical expenses per capita, GDP per capita. Then, relative risk (RR) and 95% confidence interval (CI) of PTB due to the exposure to extreme temperature were calculate in each model.

Finally, extreme temperature effect value of 14 prefecture-level cities obtained by GAMs were summarized through meta-analysis to obtain the final effect value of extreme temperature on the reported morbidity of PTB. The fixed-effect model or random-effect model was selected based on the result of heterogeneity test. Heterogeneity test was assessed using Cochran’s *Q* statistic and *I*^*2*^ value.

Statistical analyses mentioned above were implemented with the use of R (version 3.6.3, R Foundation for Statistical Computing, Vienna, Austria), SPSS 22.0 (SPSS Inc., Chicago, IL, USA) and Stata 16.0 (StataCorp LP, College Station, Texas, USA). GAMs in our analysis were performed using R with the “mgcv” package. Statistical significance was defined as the statistical tests were two-sided, and *p* value less than 0.05.

## Results

### Descriptive analysis for the PTB

**Fig. 1:**
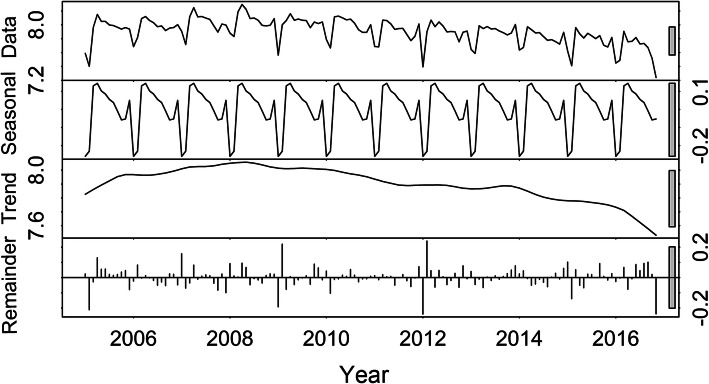
Seasonal decomposition of PTB in Shandong Province from 2005 to 2016

**Fig. 2:**
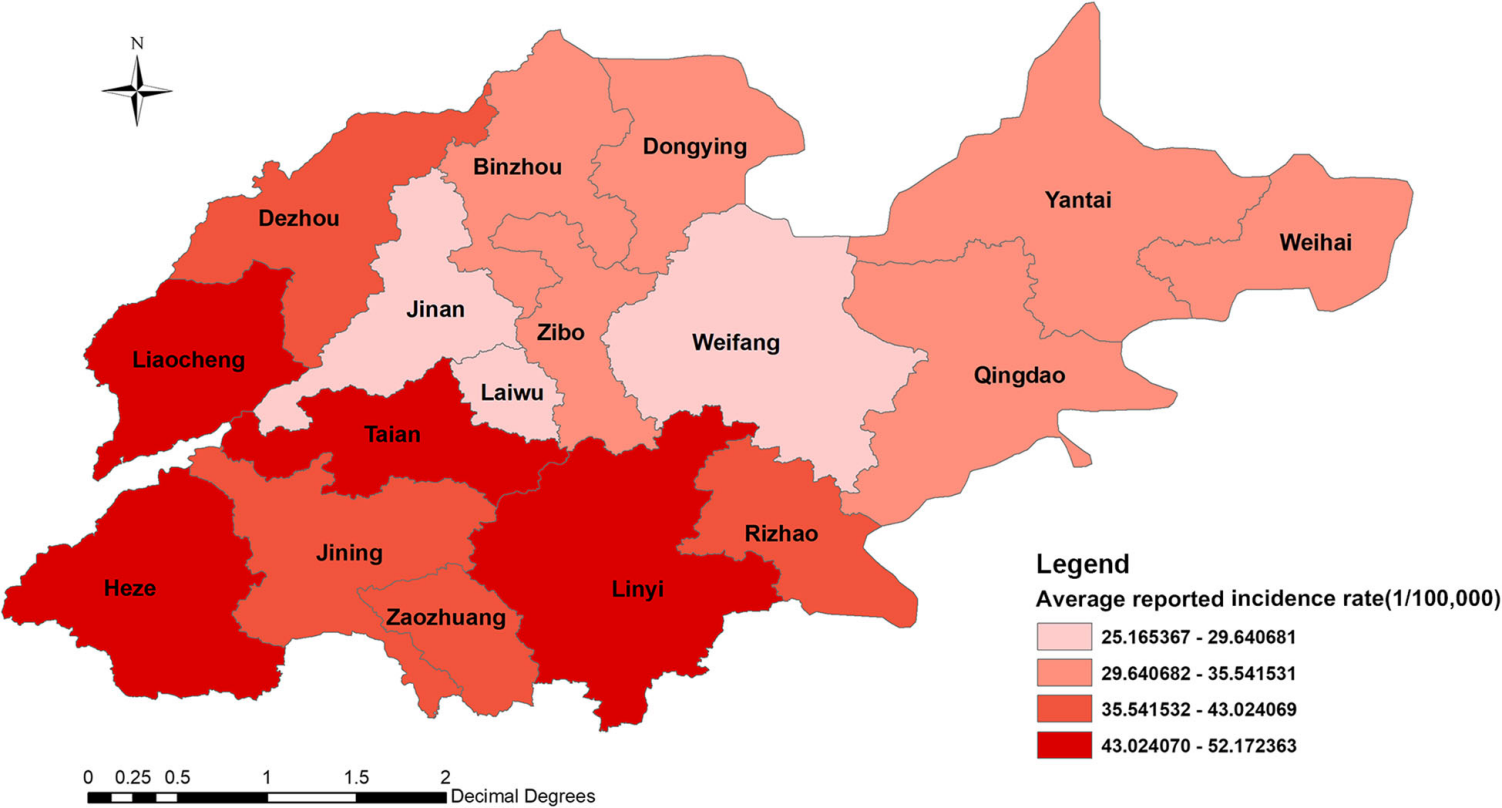
The average annual reported incidence rate of PTB in Shandong Province

During the study period, a total of 446,016 PTB cases were notified in Shandong Province. Figure 1 shows the result of the time series seasonal decomposition analysis for PTB, mainly including the raw data, seasonal variation, trend and remainder. The peak of reported cases of PTB was observed in spring. The trend of reported cases of PTB displayed increasing firstly with peaking in April 2008, and then decreasing. The spatial distribution of the average annual reported incidence rate of PTB is presented in Fig. 2. The higher reported incidence rate areas were located in Liaocheng, Taian, Linyi and Heze, while there were 3 prefecture-level cities with a lower reported incidence rate of PTB, i.e., Jinan, Laiwu and Weifang.

### Spearman correlation analysis

**Fig. 3:**
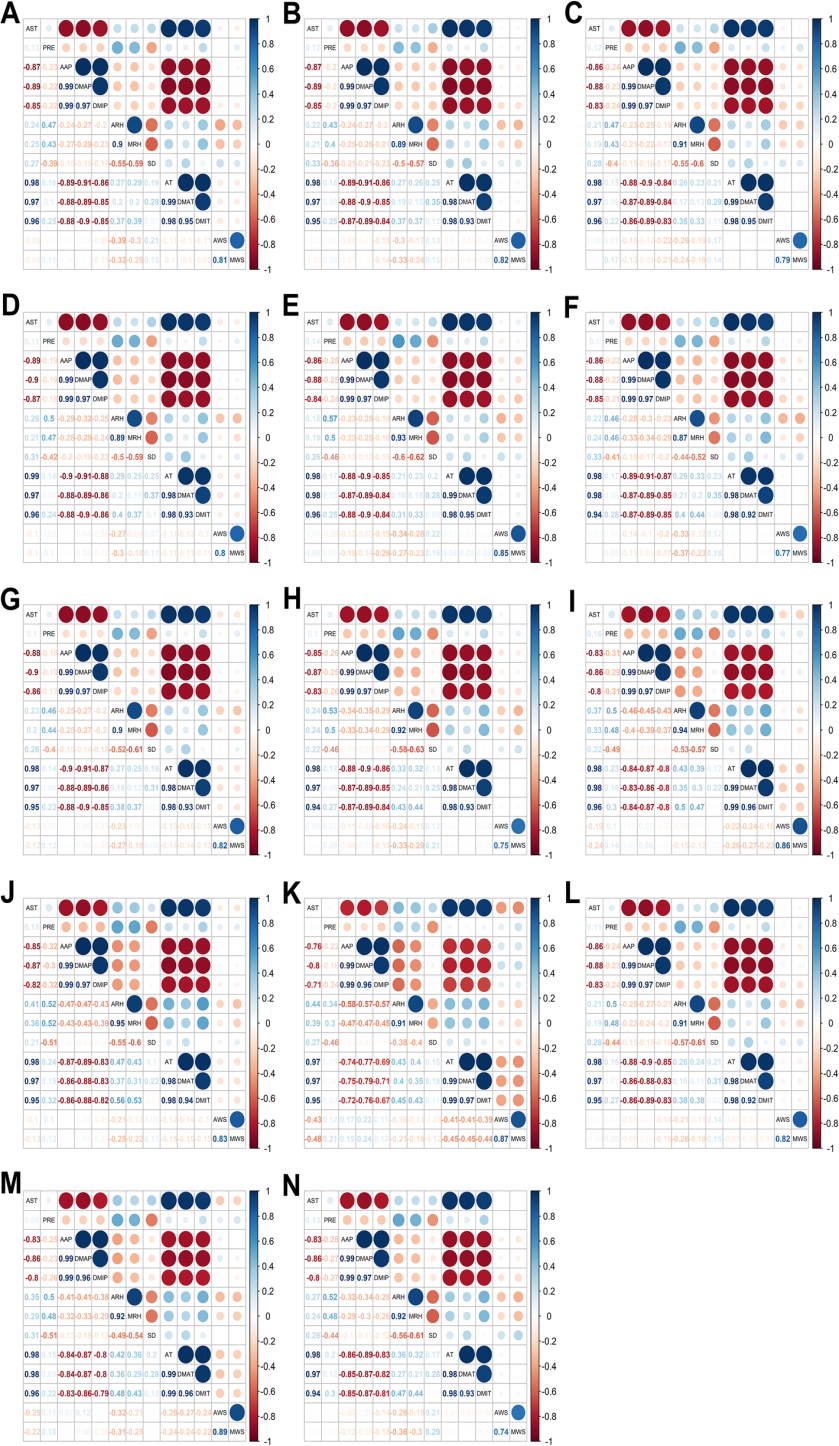
The Spearman correlation between meteorological factors in each prefecture-level city. **a** Binzhou; **b** Dezhou; **c** Dongying; **d** Heze; **e** Jinan; **f** Jining; **g** Liaocheng; **h** Linyi; **i** Qingdao; **j** Rizhao; **k** Weihai; **l** Weifang; **m** Yantai; **n** Zibo. Abbreviations: AAP, average air pressure; ARH, average relative humidity; AST, average surface temperature, AT, average temperature; AWS, average wind speed; DMAP, daily maximum air pressure; DMAT, daily maximum temperature; DMIP, daily minimum air pressure; DMIT, daily minimum temperature; MAWS, maximum wind speed; MIRH, minimum relative humidity; PRE, precipitation; SD, sun duration

Figure 3 shows the Spearman correlation between meteorological factors in each prefecture-level city. In 14 prefecture-level cities, AST, AAP, DMAP, DMIP, AT, DMAT and DMIT were strongly correlated with each other (|*r*| > 0.7). Therefore, above meteorological variables were not used in the next GAMs due to multi-collinearity. Strong correlations were detected between ARH and MRH, between AWS and MWS, with the absolute value of *r* lager than 0.7. The effects of AWS and ARH on PTB had already been demonstrated in previous studies [19, 22], so AWS and ARH rather than MRH and MWS were incorporated in the next GAMs. Finally, four independent meteorological variables were selected in the cross-correlation analysis and GAMs, including PRE, SD, AWS and ARH.

### Cross-correlation analysis

The lag time of each meteorological factor was determined according to the maximum effective CCF. The obtained results of cross-correlation analysis between daily reported incidence rate of PTB and meteorological variables are summarized in Table 1. The lag time of AT was regarded as the lag time of extreme temperature events. These lagged effects were incorporated in the next GAMs.

**Table 1:** Cross-correlation between meteorological factors and reported incidence rate of PTB in 14 prefecture-level cities, 2005–2016

City	PRE			ARH			SD			AWS			AT		
	Lag	CCF	SE	Lag	CCF	SE	Lag	CCF	SE	Lag	CCF	SE	Lag	CCF	SE
Binzhou	0	−0.036	0.015	26	−0.044	0.015	1	0.077	0.015	3	0.110	0.015	88	−0.057	0.015
Dezhou	67	−0.058	0.015	62	−0.076	0.015	26	0.052	0.015	10	0.074	0.015	86	−0.098	0.015
Dongying	70	−0.043	0.015	76	−0.065	0.015	0	0.073	0.015	1	0.116	0.015	90	−0.081	0.015
Heze	0	−0.053	0.015	37	0.043	0.015	0	0.051	0.015	86	−0.063	0.015	87	−0.069	0.015
Jinan	10	0.039	0.015	57	−0.043	0.015	5	0.074	0.015	17	0.085	0.015	3	0.101	0.015
Jining	8	0.041	0.015	68	−0.106	0.015	56	−0.056	0.015	18	0.122	0.015	0	0.108	0.105
Liaocheng	44	0.038	0.015	87	0.039	0.015	6	0.053	0.015	47	−0.038	0.015	0	0.048	0.015
Linyi	26	−0.031	0.015	83	−0.066	0.015	2	0.068	0.015	11	0.082	0.015	85	−0.092	0.015
Qingdao	18	0.052	0.015	3	0.057	0.015	47	0.057	0.015	87	0.071	0.015	5	0.104	0.015
Rizhao	32	0.037	0.015	84	−0.071	0.015	0	0.05	0.015	36	0.044	0.015	83	−0.086	0.015
Weihai	79	−0.041	0.015	16	0.087	0.015	1	0.053	0.015	88	0.084	0.015	90	−0.156	0.015
Weifang	9	0.046	0.015	89	−0.051	0.015	26	0.058	0.015	45	0.092	0.015	0	0.100	0.015
Yantai	10	0.054	0.015	89	−0.088	0.015	1	0.074	0.015	81	0.080	0.015	88	−0.175	0.015
Zibo	65	−0.035	0.015	77	−0.062	0.015	29	0.060	0.015	21	0.059	0.015	84	−0.056	0.015

Abbreviations: *ARH* average relative humidity, *AT* average temperature, *AWS* average wind speed, *CCF* cross-correlation coefficient, *PRE* precipitation, *SD* sunshine duration, *SE* standard error

### GAM and meta-analysis

**Fig. 4:**
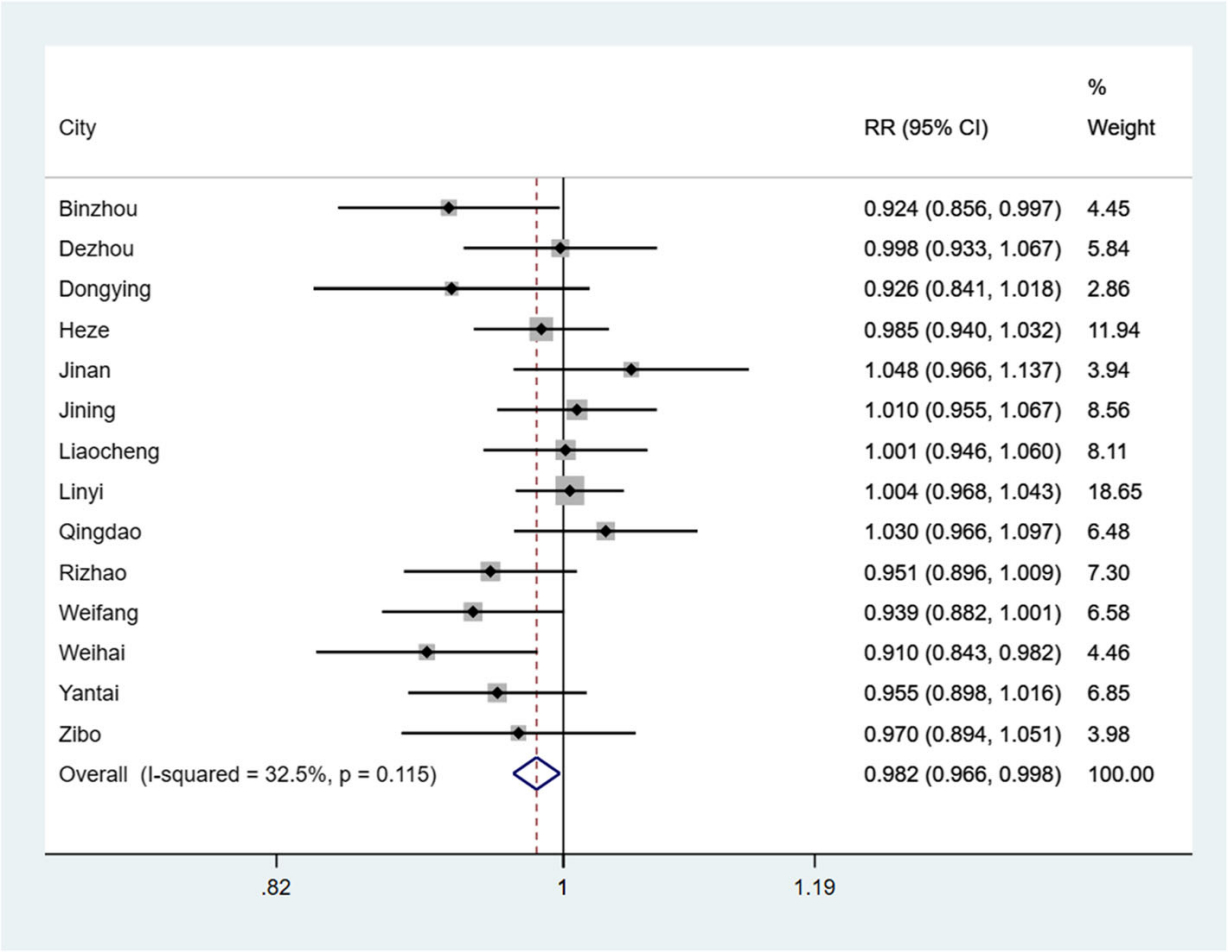
The pooled analysis and the association between extreme high temperature and daily reported incidence rate of PTB in 14 prefecture-level cities in Shandong Province

**Fig. 5:**
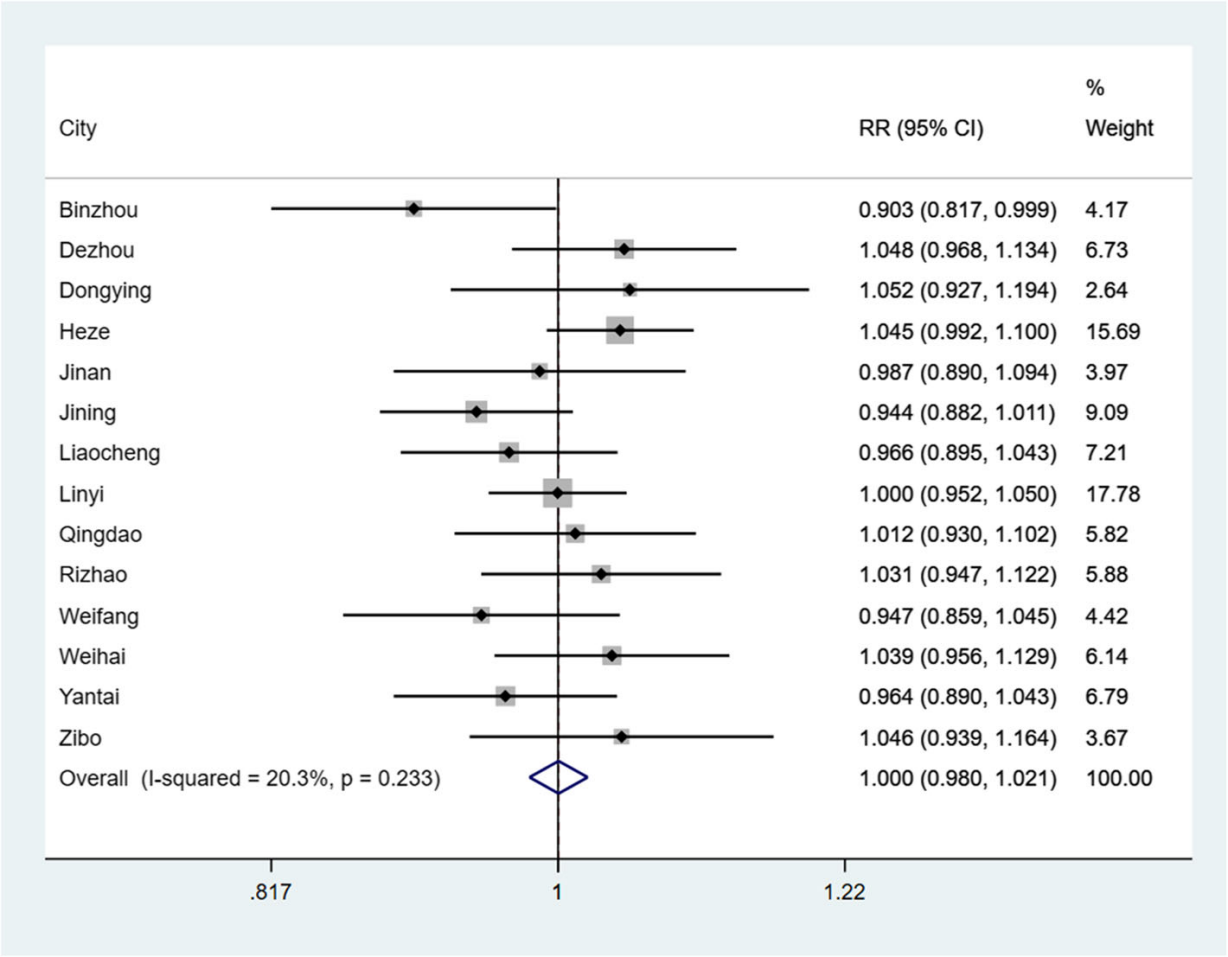
The pooled analysis and the association between extreme low temperature and daily reported incidence rate of PTB in 14 prefecture-level cities in Shandong Province

RRs of extreme temperature on the risk of PTB in each GAM and pooled results are presented in Figs. 4 and 5. After controlling for long-term effects, seasonal trends, SD, PRE, ARH, AWS, annual medical expenses per capita and GDP per capita, 14 GAMs were adopted to quantify the relationship between extreme temperature and PTB. GAMs analysis showed that extreme low temperature had a positive effect on reported morbidity of PTB (RR = 0.903, 95% CI: 0.817–0.999) in Binzhou. In Binzhou and Weihai, extreme high temperature was also found to have a positive effect on reported morbidity of PTB, with RR were 0.924 (95% CI: 0.856–0.997) and 0.910 (95% CI: 0.843–0.982), respectively. As shown in Fig. 4, although the association between extreme high temperature and PTB were found in only two areas, extreme high temperature was still associated with a decreased risk of reported morbidity of PTB (RR = 0.982, 95% CI: 0.966–0.998) in the meta-analysis. However, the association between extreme low temperature and PTB was not statistically significant in the meta-analysis (See Fig. 5). In the sensitivity analysis, no individual area substantially influenced the pooled RRs for both high temperature and low temperature (See Supplementary Figs. S1–S2, Additional File 1).

## Discussion

To our knowledge, this was the first multi-city study to examine the association between extreme temperature events and PTB using a mixed method in Shandong Province. The pooled results confirm that exposure of extreme high temperature was positively associated with decreased risk of daily reported incidence rate of PTB, and no association was found between extreme low temperature and reported morbidity of PTB. Therefore, it is important to equip policymakers with information on the effect of extreme temperature events on PTB to help guide the provision, planning and optimization of PTB control strategies in Shandong Province.

The study has confirmed that the reported morbidity of PTB presents seasonal trend, with a peak during the spring of every year (from March to May). Similar seasonal trends of PTB has been reported elsewhere [23, 24]. However, as found by Liao et al. [25], the peak of tuberculosis reported rate was between January and March in Chongqing, China. One study from Mexico reported that the tuberculosis has seasonality with spring and summer being the peak seasons [26]. The seasonality of reported morbidity of PTB may be associated with suitable environment conditions that can affect the immune system and facilitate the dispersion of the pathogen. Therefore, the above seasonal differences are considered to be related to geographical and demographic factors [27]. In order to cope with the arrival of the incidence peak period and increase awareness-raising, resources should be distributed and planned for rationally during this period.

In our study, the lagged effects of temperature on the reported morbidity of PTB in different prefecture-level cities are different. Some studies have evaluated that there is a potential lagged effect of the temperature on PTB [19, 28, 29]. A study of Qinghai in China showed that the effect of temperature on the log of tuberculosis incidence was a delay of 3 months [19]. A study from Bangladesh found that the significant associations between temperature and morbidity of tuberculosis were proved at lag periods up to 6 quarters [28]. Another study from China estimated that temperature 2 months ahead was related with morbidity of tuberculosis in the current month [29]. This discrepancy in lag time of temperature on PTB may be due to different study locations, different social-economic status and health sources, or different research methods, etc. For example, different sites may reveal different temperature patterns [30].

We adopted a mixed method-GAM and meta-analysis, which can estimate the relationship between extreme temperature events and PTB at city and provincial levels. In this study, we found that extreme high temperature was associated with a significant decrease in the risk of PTB at provincial level, although only two prefecture-level cities had found an effect of extreme high temperature on PTB. Epidemiological study has demonstrated that extreme temperature could affect the risk of mortality [31, 32], acute myocardial infarction hospital admissions [33], cardiovascular emergency hospitalizations [34], injury [35], infectious diseases (such as, mumps [10], hand-foot-mouth disease [11, 36]), etc. In recent years, temperature has been acknowledged as a significant influence factor related to PTB [19, 28, 29]. A study by Rao et al. [19] suggested that each 10 °C increase in temperature, associated with 9% decrements in the morbidity of tuberculosis. Additionally, studies of Kuddus et al. [28] and Xiao et al. [29] were in accordance with the findings of our study. The biological mechanism linking temperature and PTB is complex. Firstly, ultraviolet radiation is high during extreme high temperature, and it can be used to protect the spread of PTB [37]. Decreasing PTB may be explained by reductions in the *Mycobacterium tuberculosis* of air driven by increasing ultraviolet radiation. Secondly, higher temperature may change human habits and behaviors [38]. Under extreme high temperature, people are more likely to stay in air-conditioned environments, which decrease the risk of PTB attributed to close contact. Simultaneously, close contact of indoor crowding and warm environment are not only conducive to the survival of *Mycobacterium tuberculosis*, but also increase the possibility of PTB transmission in winter [39]. Furthermore, vitamin D plays an important role in progression to active tuberculosis disease [40], however, vitamin D is easily deficient in winter and may lead to declined immunity [41]. In turn, the above two explanations which supports our opinion, namely, extreme high temperature may be a protective factor in the development of PTB. However, this result is inconsistent with the finding of Onozuka et al. [42], Zhang et al. [43] and Tian et al. [44]. In addition, our study did not find effect of extreme low temperature events on the reported morbidity of PTB at provincial level. On the hand, differences may be due to different study regions and methods. On the other hand, Shandong Province is generally characterized by a warm temperate monsoon and is not a high incidence area of PTB [45], which may be the reasons for the inconsistency with other studies.

Some limitations of our study should be acknowledged. Firstly, due to deficiency of meteorological data, three prefecture-level cities of Shandong Province were not included in our study. Secondly, the reported PTB data were from the China Information System for Disease Control and Prevention, and under-reporting bias is inevitable. Thirdly, some confounders like pathogen mutation, individual behaviors, and immune levels have not been considered to control. Hence, further more studies are required to demonstrate the possible associations between extreme temperature and PTB.

## Conclusion

The results of our study provide evidence that extreme high temperature decrease the risk of reported morbidity of PTB. This study supplied helpful cognition for targeted PTB control and prevention associated with extreme temperature in Shandong Province. Meanwhile, our findings provide more evidence that it is necessary to consider the effect of extreme temperature on human health, and develop strategies to response to climate change.

## Supplementary Information

**Additional file 1: Figure S1.** Sensitivity analysis of the overall pooled extreme high temperature study. **Figure S2.** Sensitivity analysis of the overall pooled extreme low temperature study.

## Data Availability

The datasets used during the current study are available from the corresponding author on reasonable request (dgy-153@163.com). Demographic and meteorological data are obtained by the following links: http://www.phsciencedata.cn/Share/ and http://data.cma.cn/. And these data are publicly available only to core users.

## Abbreviations

AAP: Daily average air pressure
ARH: Daily average relative humidity
AST: Daily average surface temperature
AT: Daily average temperature
AWS: Daily average wind speed
CCF: Cross-correlation coefficient
CI: Confidence interval
DMAP: Daily maximum air pressure
DMAT: Daily maximum temperature
DMIP: Daily minimum air pressure
DMIT: Daily minimum temperature
GAM: Generalized additive model
GCV: Generalized cross-validation
GDP: Gross domestic product
IPCC: The Intergovernmental Panel on Climate Change
MRH: Daily minimum relative humidity
MWS: Daily maximum wind speed
PRE: Daily precipitation
PTB: Pulmonary tuberculosis
RR: Relative risk
SD: Sunshine duration
SE: Standard error
