# Supplementary material for: The association between extreme temperature and pulmonary tuberculosis in Shandong Province, China, 2005–2016: a mixed method evaluation
Source: BMC Infect Dis. 2021 May 1;21:402. doi: 10.1186/s12879-021-06116-5 (PMC8088045; doi:10.1186/s12879-021-06116-5)
Supplement: Supplementary file 1 — Additional file 1: Figure S1. Sensitivity analysis of the overall pooled extreme high temperature study. Figure S2. Sensitivity analysis of the overall pooled extreme low temperature study. [file 12879_2021_6116_MOESM1_ESM.docx]

**SUPPLEMENTARY INFORMATION**

**The association between extreme temperature and pulmonary tuberculosis in Shandong Province, China, 2005-2016: a mixed method evaluation.**

**Dongzhen Chen^1#^, Hua Lu^2#^, Shengyang Zhang^3#^, Jia Yin^1#^,** **Xuena Liu^1^, Yixin Zhang^3^, Bingqin Dai^3^, Xiaomei Li^1*^, Guoyong Ding^1*^**

1 School of Public Health, Shandong First Medical University & Shandong Academy of Medical Sciences, 271016, Taian, Shandong Province, China

2 Taian Centers for Diseases Prevention Control, 271000, Taian, Shandong Province, China

^3^ Shandong Center for Disease Control and Prevention, 250014, Jinan, Shandong Province, China

^#^These authors contributed equally for this work.

^*^Corresponding Authors (Guoyong Ding and Xiaomei Li)

**Figure S1**. Sensitivity analysis of the overall pooled extreme high temperature study.

**Figure S2**. Sensitivity analysis of the overall pooled extreme low temperature study.

**Figure S1**. Sensitivity analysis of the overall pooled extreme high temperature study.

**Figure S2**. Sensitivity analysis of the overall pooled extreme low temperature study.
